# Harnessing the Potential of Native Microbial Communities for Bioremediation of Oil Spills in the Iberian Peninsula NW Coast

**DOI:** 10.3389/fmicb.2021.633659

**Published:** 2021-04-23

**Authors:** Maria L. Bôto, Catarina Magalhães, Rafaela Perdigão, Diogo A. M. Alexandrino, Joana P. Fernandes, Ana M. Bernabeu, Sandra Ramos, Maria F. Carvalho, Miguel Semedo, Julie LaRoche, C. Marisa R. Almeida, Ana P. Mucha

**Affiliations:** ^1^Bioremediation and Ecosystems Functioning (EcoBioTec), CIIMAR – Interdisciplinary Centre of Marine and Environmental Research, University of Porto, Porto, Portugal; ^2^Institute of Biomedical Sciences Abel Salazar (ICBAS), University of Porto, Porto, Portugal; ^3^Faculty of Sciences (FCUP), University of Porto, Porto, Portugal; ^4^Ocean Frontier Institute, Dalhousie University, Halifax, NS, Canada; ^5^Marine and Environmental Geology (GEOMA) Group, Department of Marine Geosciences, University of Vigo, Vigo, Spain; ^6^Department of Biology, Dalhousie University, Halifax, NS, Canada

**Keywords:** bioremediation, oil spills, georeferenced library, native microorganisms, next-generation sequencing, predictive functional profiling, enrichment experiments

## Abstract

Oil spills are among the most catastrophic events to marine ecosystems and current remediation techniques are not suitable for ecological restoration. Bioremediation approaches can take advantage of the activity of microorganisms with biodegradation capacity thus helping to accelerate the recovery of contaminated environments. The use of native microorganisms can increase the bioremediation efficiency since they have higher potential to survive in the natural environment while preventing unpredictable ecological impacts associated with the introduction of non-native organisms. In order to know the geographical scale to which a native bioremediation consortium can be applied, we need to understand the spatial heterogeneity of the natural microbial communities with potential for hydrocarbon degradation. In the present study, we aim to describe the genetic diversity and the potential of native microbial communities to degrade petroleum hydrocarbons, at an early stage of bioremediation, along the NW Iberian Peninsula coast, an area particularly susceptible to oil spills. Seawater samples collected in 47 sites were exposed to crude oil for 2 weeks, in enrichment experiments. Seawater samples collected *in situ*, and samples collected after the enrichment with crude oil, were characterized for prokaryotic communities by using 16S rRNA gene amplicon sequencing and predictive functional profiling. Results showed a drastic decrease in richness and diversity of microbial communities after the enrichment with crude oil. Enriched microbial communities were mainly dominated by genera known to degrade hydrocarbons, namely *Alcanivorax*, *Pseudomonas*, *Acinetobacter*, *Rhodococcus*, *Flavobacterium*, *Oleibacter*, *Marinobacter*, and *Thalassospira*, without significant differences between geographic areas and locations. Predictive functional profiling of the enriched microbial consortia showed a high potential to degrade the aromatic compounds aminobenzoate, benzoate, chlorocyclohexane, chlorobenzene, ethylbenzene, naphthalene, polycyclic aromatic compounds, styrene, toluene, and xylene. Only a few genera contributed for more than 50% of this genetic potential for aromatic compounds degradation in the enriched communities, namely *Alcanivorax*, *Thalassospira*, and *Pseudomonas* spp. This work is a starting point for the future development of prototype consortia of hydrocarbon-degrading bacteria to mitigate oil spills in the Iberian NW coast.

## Introduction

Each year, three million tons of oil are released into the sea due to leaks or accidental oil spills resulting from the exploration, production, refining, transportation and storage of petroleum and its derivatives (Brooijmans et al., 2009; Das and Chandran, 2011). Oil spill accidents, in particular, have serious impacts in all ecosystems (Cerqueda-García et al., 2020) and the high concentration of the toxic chemicals released after an oil spill persist in the environment for years to come, which highlights the need for future prevention planning.

The effectiveness of a response to mitigate an oil spill depends on the oil type, location, and the spill size (Etkin, 2000). Current remediation approaches are expensive, have a short-term effect and do not allow a complete ecological restoration (Etkin, 2000; Mullin and Champ, 2003). A more effective approach to mitigate oil spills is to transform the pollutants into less toxic substances or to completely degrade them (González, 2011). Bioremediation, in this context, is an approach that uses the natural ability of microorganisms to detoxify/degrade petroleum compounds. This approach ensures the mineralization of the oil and avoids the risk of recontamination with secondary contaminants (Fuentes et al., 2014). Since bioremediation can be helpful to ensure ecological restoration and to accelerate hydrocarbon (HC) degradation, this methodology can be considered to be an environmentally friendly, cost-effective and efficient tool to mitigate oil polluted sites (Hosokawa et al., 2009; Pontes et al., 2013; Gouveia et al., 2018; Perdigão et al., 2021).

Crude-oil is a heterogeneous mixture of more than 17000 complex chemical components with predominance of hydrocarbons (Brooijmans et al., 2009). Due to their complex composition, volatility, wide distribution and bioavailability, HCs are one of the most persistent organic pollutants in every environment (Fuentes et al., 2014). Hydrocarbons are generally biodegraded in order of increasing complexity from lower molecular weight HCs (linear alkanes) to higher ones (like cyclic alkanes or aromatic HC) starting from n-alkanes to branched-chain alkanes, branched alkenes, low-molecular-weight n-alkyl aromatics, monoaromatics, cyclic alkanes, PAHs, and then asphaltenes (Tyagi et al., 2011). Therefore, the susceptibility to biodegrade an HC compound depends on its structure (Das and Chandran, 2011).

Petroleum degrading microorganisms can be classified into obligate hydrocarbonoclastic bacteria (OHCB), that have the ability to grow on a narrow range of HCs and HCs degradation products, such as the genera *Alcanivorax*, *Cycloclasticus*, *Marinobacter*, *Oleiphilus*, *Oleispira*, *Thallassolituus*, and generalist microorganisms that can grow on a wider range of carbon sources (Yakimov et al., 2007; Ron and Rosenberg, 2014; Mapelli et al., 2017). Numerous prokaryotes are capable of degrading n-alkanes (e.g., *Acinetobacter*, *Alcanivorax, Nocardia, Oleiphilus, Prauserella, Pseudomonas*, and *Rhodococcus*) (Fuentes et al., 2014), however, as the complexity of the HC molecular structure increases, the number of organisms capable of degrading them decreases (Varjani, 2017). In fact, the type of oxidizing reactions that occur during the degradation pathways of different petroleum HCs varies accordingly to the specific oxygenases and dioxygenases found in different microorganisms (Bacosa et al., 2018). Since different organisms can degrade different petroleum compounds, a concerted action of a microbial consortium is expected to achieve a better bioremediation performance by degrading a larger diversity of HCs through the combination of the genetic machinery of more than one microorganism (Sathishkumar et al., 2008; Li et al., 2016). Previous studies indicate that the best results for HC removal were obtained when indigenous microorganisms, pre-adapted to degrade oil compounds, were used in an oil spill accident simulation (Nikolopoulou et al., 2013). Therefore, indigenous microorganisms can be an advantageous strategy to enhance HC degradation thus avoiding the unpredictable ecological impacts that may result from the introduction of exogenous microorganisms (Tyagi et al., 2011; Gouveia et al., 2018).

Traditional methodology for assessing hydrocarbon-degrading bacteria rely on cultivation-dependent approaches, which underestimate the vast biodiversity present in the natural world (Hugenholtz, 2002; Fuentes et al., 2014). Moreover, this constraint prevented scientists to reproduce natural environments in synthetic/artificial media and to fully understand the real highly complex and interactive individuals of multi-assemblages (Joint et al., 2010). Culture-independent approaches based on molecular techniques allowed insight into the structure, composition and dynamics of natural hydrocarbon-degrading bacteria (Chikere et al., 2019). For example, when the Deepwater Horizon accident occurred, culture-independent approaches were used to follow the succession of taxa in the water column. Results showed that *Oceanospirillales* and *Pseudomonads* appeared first followed by *Colwellia*, *Cycloclasticus*, methylotrophic organisms and *Flavobacteria*, *Rhodobacteraceae*, and *Alteromonadaceae* (Redmond and Valentine, 2012; Kimes et al., 2014) appeared last. More recently, a metagenomic study conducted with cold (5°C) Norwegian seawaters in microcosms experiments amended with a mixture of seawater and chemically dispersed oil, linked the efficiency of oil degradation with succession patterns of the microbial community (Ribicic et al., 2018). This experiment showed clear successional patterns in both microbial communities and metagenome composition of genes coding for HC degrading enzymes, emphasizing that a successful substrate transformation can only be achieved by cooperation among hydrocarbon biodegrading organisms (Ribicic et al., 2018). Despite the technological breakthrough in culture-independent approaches, some NGS methods, like metagenome analysis, are still expensive. However, it is possible to predict the metabolic properties of HC-degrading microbial communities by functional inference at a high taxonomic resolution, from 16S rRNA gene sequences (Mukherjee et al., 2017; Chikere et al., 2019).

Despite the increase of recent studies in bioremediation of petroleum HCs (Chen et al., 2020; Shi et al., 2020; Perdigão et al., 2021), there is still a lack of knowledge regarding the natural repertoire of the microbial communities able to degrade petroleum HCs. This study addresses the geospatial variation of these natural microbial communities in NW coast of the Iberian Peninsula to identify prokaryotic groups with potential bioremediation applicability in this region. Furthermore, crude oil enrichment experiments allow the identification of the potential of jointed biodegrading consortium that first react to an eventual oil spill occurring within the studied geographical area, so that bioremediation approaches can be more effective in mitigating the accident.

Since the NW coast of the Iberian Peninsula is close to many trade routes of intercontinental oil transportation and is part of one of the largest oil community ports (Vieites et al., 2004), it is a suitable region for a pilot-scale investigation of native microorganisms with HC degradation potential for future development of bioremediation techniques to be implemented. For this study, two questions were posed: (i) what are the natural diversity and distribution of petroleum HC-degrading microorganisms along NW coast of the Iberian Peninsula and (ii) are enrichment experiments able to identify the microbial consortia that first responds to spill bioremediation? To address these questions, samples representing different georeferenced areas were collected along the NW coast of the Iberian Peninsula. For each sample, the initial natural prokaryotic communities were characterized and an enrichment incubation experiment was set up using petroleum as carbon source with nutrients addition to obtain enriched microbial communities. Natural and selected microbial consortia were analyzed using next-generation sequencing (NGS) of the 16S rRNA gene and generated sequences analyzed with the DADA2 and PICRUSt2 pipelines for diversity, taxonomic, and predicting functional profiling. The biodegradation potential of both natural and crude-oil enriched communities was also estimated by the Most Probable Number (MPN). In the present study, we described the genetic diversity and the potential of native microbial communities to degrade petroleum hydrocarbons, at an early stage of bioremediation, along the NW Iberian Peninsula coast, an area particularly susceptible to oil spills.

## Materials and Methods

### Study Area

**FIGURE 1:**
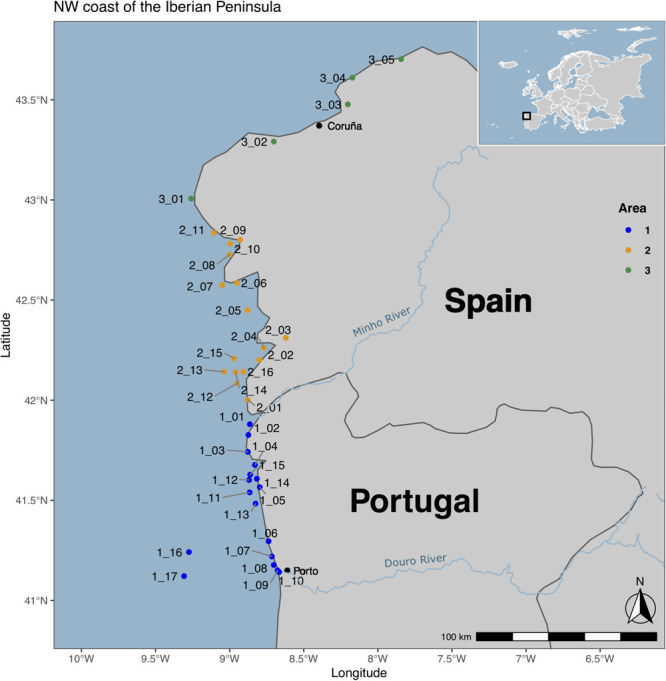
Location of the 47 sampling sites along the NW coast of the Iberian Peninsula. This map was created using R v.3.6.1 (Team, 2019), R packages ggpplot2 (Wickham, 2016), sf (Pebesma, 2018), ggspatial (Dunnington, 2020), and rnaturalearth (South, 2017). The rivers were downloaded from http://tapiquen-sig.jimdofree.com (Carlos Efraín Porto Tapiquén. Geografía, SIG y Cartografía Digital. Valencia, Spain, 2020). The map was edited using Inkscape v. 1.0.1.

This study was conducted along the NW coast of the Iberian Peninsula, from July 2017 to September 2018. Three areas were delimited: Area 1, located in the NW coast of Portugal, between the rivers Douro and Minho; Area 2, located in the NW coast of Spain, between Minho river and Finisterra Cape; and Area 3, between Finisterra Cape and Estaca de Bares cape. A total of 47 sites were sampled across the different areas (Figure 1). Water samples were collected from 10 coastal and seven offshore sites in Area 1, 11 coastal and five offshore sites from Area 2, and five coastal sites in Area 3 (Supplementary Table 1). In order to investigate temporal variations on microbial communities, seasonal sampling campaigns [Spring (Sp), Summer (Su), Autumn (Au), and Winter (Wi)] were performed in one selected station of each delimited Area, close to cargo ships routes (Ingleses beach in Area 1, Toralla beach in Area 2, and Caranza beach in Area 3).

### Sampling Procedures

For each location, 5 L of seawater were collected at the surface with a sterile container. The surface water was characterized in terms of pH, salinity, temperature, conductivity, and oxygen levels by using a multiparameter probe (YSI EXO1 Sonde). Samples were then transported to the laboratory in a refrigerated ice box. At the laboratory, samples were filtered through Sterivex^TM^ filters and processed in triplicate for crude-oil enrichment experiments and estimation of the MPN of hydrocarbon-degrading microorganisms.

All glass and plastic materials were chemically decontaminated with 10% (v/v) HCL solution and, afterward, washed with deionized water (conductivity <0.2 μS cm^–1^). The materials for microbial enrichments were sterilized by autoclaving at 121°C for 20 min and plastic materials were sterilized by UV light for 30 min.

### Preparation of Enrichment Experiments

The enrichment experiments were performed in order to select the native microbial consortia capable of degrading hydrocarbon compounds in the initial stages of the bioremediation of oil spills. The optimization of the enrichments was based on previous experiments that proved effective for microbial degradation of hydrocarbons such as those described by Almeida et al. (2013).

After seawater collection, enrichment experiments were conducted in 500 mL sterilized glass flaks, in triplicate for each site. Each flask contained 150 mL of the seawater sample supplied with 0.8 M of di-hydrogen phosphate and 1.5 M of potassium nitrate, which served as exogenous nitrogen and phosphorous sources. The only carbon source provided was Arabian light crude-oil, supplied by an oil refinery. This was added to the flasks in a proportion of 20:0.5 (v/v), after passing through a 0.2 μm sterile filter to remove any microorganisms that could contaminate our samples. The carbon from the petroleum together with the nutrients supplied formed a ratio of C/N/P of 100:10:1.

The flasks were incubated for 2 weeks under constant orbital agitation (100 rpm), at room temperature, in aerobic and aphotic conditions to avoid photo oxidation of the petroleum compounds.

After 15 days of incubation, 1 mL of medium from each triplicate flask was collected with sterile syringes to determine the abundance of hydrocarbon degraders and afterward all three replicates were combined for DNA isolation.

### Microbial Community Collection

Samples collected from the sampling sites were immediately filtered, in duplicate, through Sterivex^TM^ filters with a pore size of 0.22 μm, hydrophilic, PVDF, Durapore membrane (SVGV1010RS, Merck Millipore, Portugal) with the help of 50 mL sterile syringes, a PowerVac^TM^ Manifold (Qiagen) and a vacuum pump, following the methodologies used in the Ocean Sampling Day event (Kopf et al., 2015). The volume filtered for each replicate was approximately 2 L of seawater or the maximum volume possible in a 2-h window (ranging from 0.920 to 2 L). Then, the remaining liquid in the filters was removed by using a syringe and the Sterivex^TM^ was capped with the Inlet and Outlet caps, placed in sterile bags properly identified and stored in a −80°C freezer.

For the samples from the enrichments with crude-oil, the composite sample of the three replicates was also filtered using the same process described for natural samples. Due to the thick consistency of crude oil, the maximum volume possible was filtered (ranging from 7.0 to 181.5 mL), in a 2-h time frame or until the Sterivex^TM^ units clogged. Afterward, the remaining liquid in the filters was removed by using a syringe and the Sterivex^TM^ was capped with the Inlet and Outlet caps, placed in sterile bags properly identified and stored in a −80°C freezer.

### DNA Extraction, DNA Quantification, PCR Library Preparation and Sequencing of 16S rDNA Amplicon

Sterivex^TM^ filters resultant from filtration of natural and enriched communities were used for DNA extraction. DNA was extracted with the DNeasy^®^ PowerWater^®^ Sterivex Kit (QIAGEN, Inc.) and quantified fluorometrically using the Qubit dsDNA HS Assay (Thermo Fisher Scientific, Waltham, MA, United States). The amplification of the V4–V5 region of the genetic marker 16S rRNA gene was performed by using the primer pair 515YF (5′-GTGYCAGCMGCCGCGGTAA-3′) and Y926R-jed (5′-CCGYCAATTYMTTTRAGTTT-3′), according to Earth Microbiome Project protocols (Gilbert et al., 2014). These primers were adapted from the primer pair 515F/806R, that only amplified the V4 region, designed by Caporaso et al. (2011, 2012), and latter modified by Apprill et al. (2015) and Parada et al. (2016).

Sequencing of 16S rRNA gene amplicons was performed in Biocant – Biotechnology Park (Cantanhede, Portugal). Two Polymerase Chain Reaction (PCR) rounds were performed to amplify first the DNA with the specific primers and reamplify afterward to add sequencing adapters and dual indexes. The KAPA HiFi HotStart PCR kit was used to perform the PCR reactions, which included 0.3 μM of each primer and 12.5 ng of template DNA in a total volume of 25 μL. Conditions of PCR involved denaturation at 95°C for 3 min followed by 25 cycles of 20 s at 98°C, 30 s at 50°C, 30 s at 72°C with a final extension for 5 min at 72°C. The second round of PCR reactions were performed according to the manufacturer’s suggestions (Illumina, 2013). At Genoinseq (Cantanhede, Portugal), the PCR products were one-step purified and normalized by using the SequalPrep 96-well plate kit (Thermo Fisher Scientific, Waltham, MA, United States) (Comeau et al., 2017), pooled and pair-end sequenced, according to the manufacturer’s instructions, in the Illumina MiSeq^®^ sequencer with the V3 chemistry (Illumina, San Diego, CA, United States). The raw Illumina fastq files for this study were deposited in the European Nucleotide Archive (ENA) at EMBL-EBI under accession number PRJEB43289^1^.

### Hydrocarbon Degraders Abundance

For this work, the Most Probable Number (MPN) procedure was adapted from Wrenn and Venosa (1996) to determine the abundance of hydrocarbon degrading bacteria in natural samples and enriched samples. This method was performed, in triplicate, in 96-well microtiter plates using the growth medium Bushnell Haas medium supplemented with 2% NaCl. To determine the total HC degraders, the selective substrate was pre-filtered (0.2 μm) Arabian light crude-oil, according to the description available in Gouveia et al. (2018). The abundance of HC degrading microorganisms was then estimated using the Most Probable Number Calculator v3.1 program (Klee, 1993). The MPN method was applied to natural and enriched communities.

### Bioinformatics Pipeline

#### Microbial Community Analysis With QIIME

The QIIME2 (Quantitative Insights Into Microbial Ecology) bioinformatic platform was used to pre-process, merge, filter by quality and to apply the DADA2 pipeline (Caporaso et al., 2010; Bolyen et al., 2019).

Since the raw data provided by Genoinseq (Cantanhede, Portugal) was already demultiplexed, the first step taken in QIIME2 pipeline was to remove the forward and reverse primers of each amplicon by using cutadapt with default parameters (Martin, 2011). Afterward, a quality control of the high throughput fastq data was assessed through graphics and plots by using the java-based software FastQC, version 0.11.9 (Andrews, 2010). This step was important to assure that the raw data had no problems or biases originated in both the sequencer and the starting library material. Then the DADA2 pipeline was used to filter reads and chimeras, join paired-end reads, denoise and dereplicate sequences and providing an Amplicon Sequence Variance (ASV) table as output (Callahan et al., 2016). In this step, the trim for the forward and reverse sequences were, respectively, 270 and 220 to maintain a high Q score and sequence overlap. The number of reads to train the error model were 30000. For taxonomic assignment, the ASVs representative sequences were classified with the Naïve Bayes classifier using Silva reference database version 132 (available at https://www.arb-silva.de/download/archive/qiime) (Yilmaz et al., 2014). The Naïve Bayes classifier was chosen due to the training accuracy of this classifier being improved by the hypervariable region targeted by the primers used in this study. Afterward, the taxa Eukaryota, Chloroplast and Mitochondria were removed from the final ASV table with taxonomy.

#### Predictive Functional Profiling (PICRUSt2)

The prediction of the functional composition of the metagenomes was accomplished by using PICRUSt2 (Phylogenetic Investigation of Communities by Reconstruction of Unobserved States) (version v2.3.0 beta) (Douglas et al., 2019). The absolute abundance table of ASVs, obtained from DADA2, was used as input and the pipeline was run using the default parameters.

For this work, a total of 397 KEGG orthologs (KOs) were identified and analyzed for ten hydrocarbon degradation pathways: Aminobenzoate degradation (00627) (with a total of 53 different KOs), Benzoate degradation (00362) (82 KOs), Chlorocyclohexane and chlorobenzene degradation (00361) (26 KOs), Degradation of aromatic compounds (01220) (98 KOs), Ethylbenzene degradation (00642) (9 KOs), Naphthalene degradation (00626) (15 KOs), Polycyclic aromatic hydrocarbon degradation (00624) (34 KOs), Styrene degradation (00643) (18 KOs), Toluene degradation (00623) (33 KOs), and Xylene degradation (00622) (29 KOs) (Kanehisa and Goto, 2000; Kanehisa et al., 2018; Kanehisa, 2019).

In order to analyze the contributions of the different ASV for each metabolism, the *--stratified* option was added to the picrust2 full pipeline (*picrust2_pipeline.py*). For each degradation pathway (group of KOs), the absolute contribution of each ASV for each KO (“taxon_function_abun” column in the stratified output table) was added at the genus level and divided by the total contributions of that group of KOs using the *group_by* and *summarize* functions of the dplyr R package (v. 0.8.3) (Wickham et al., 2019b). Unclassified ASVs at the genus level were agglomerated by phylum, i.e., all unclassified ASVs of each phylum were counted as a single genus. The relative contribution in each sample was then averaged for natural and enriched communities.

### Data and Statistical Analysis

To assess if the sequencing effort represented the full microbial diversity present in all samples, rarefaction curves were performed for all samples. Alpha diversity indexes, such as species richness, Shannon diversity, and Berger-Parker were estimated after rarefying all samples to the lowest number of reads (*n* = 16762), using absolute ASV numbers agglomerated at genus level, using the vegan package (v. 2.5-6) (Oksanen et al., 2019) and diverse package (v.0.1.5) (Guevara et al., 2016) in R (v 3.6.1) (Team, 2019). These indices allowed to characterize the spatial and temporal diversity of prokaryotic communities in the studied areas and also in the enriched samples. The graphs were made using the R packages ggplot2 (v. 3.2.1) (Wickham, 2016), scales (v. 1.1.0) (Wickham and Seidel, 2019), dplyr (v. 0.8.3) (Wickham et al., 2019b), ggpubr (v.0.2.4) (Kassambara, 2019), gridExtra (v. 2.3) (Auguie, 2017), and cowplot (v. 1.0.0) (Wilke, 2019).

Regarding the beta diversity analysis, a hierarchical clustering of the microbial communities was performed with the R package vegan (v.2.5-6) (Oksanen et al., 2019) and the NMDS analysis was performed using the packages vegan (v. 2.5-6) (Oksanen et al., 2019), phyloseq (v 1.30.0) (McMurdie and Holmes, 2013), and ggplot2 (v3.2.1) (Wickham, 2016). Both analyses were performed using absolute ASV numbers agglomerated at genus level and rarefying all samples to the lowest number of reads (*n* = 16762), which allowed to evaluate the structure of the microbial communities in natural and enriched conditions. To evaluate the similarities between all natural and oil-enriched communities, an analysis of similarities (ANOSIM) was performed using PRIMER 6 software (Clarke and Gorley, 2006).

The taxonomic analyses at both phylum and genus levels were done with R phyloseq package (v. 1.30.0) (McMurdie and Holmes, 2013) and the corresponding graphics were made using the tidyverse package (v. 1.3.0) (Wickham et al., 2019a) in R.

A generalist and specialist taxa analysis based on the presence of absolute abundance of ASVs was also performed for natural and enriched microbial communities to distinguish which group of genera were present in both communities and which groups were more abundant in each community. To do so, an ASV table with the sum of absolute ASVs abundances agglomerated at genus level for natural and enriched communities was used. Both communities were then rarefied to the lowest sum of reads (*n* = 1624619) and the clam command in vegan package (v. 2.5-6) (Oksanen et al., 2019), in R, was used. The output table contained the sum of each ASV (without taxonomy) present in the natural and enriched communities, as well as the type of specialization attributed to each ASV. Afterward, the corresponding taxonomic classification was matched to the respective ASV using the “vlookup” function in Excel.

## Results

### Upstream Sequence Outputs

Sequencing analysis yielded a total of 6266779 raw sequences, which after the DADA2 pipeline, decreased to 3867589. The number of raw reads recovered from the natural community DNA samples varied between 42759 and 114733 and the number of raw reads recovered from samples after the petroleum enrichments varied between 31639 and 108914 (Supplementary Table 2). After bioinformatics analysis, the total number of sequences present in natural communities ranged between 20793 and 76669 and the total number of sequences in enriched communities ranged between 16762 and 70878.

### Shifts in Prokaryotic Diversity

#### Alpha and Beta Diversity

**FIGURE 2:**
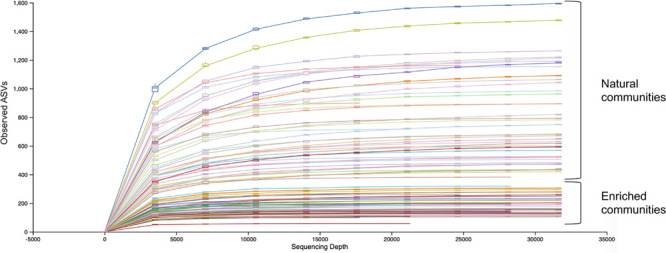
Alpha rarefaction curves of natural and enriched microbial communities of 47 sampling sites.

Rarefaction curves (Figure 2) show that the sequencing effort captured the entire diversity present in both natural and enriched microbial communities, as the curves have reached a plateau.

**FIGURE 3:**
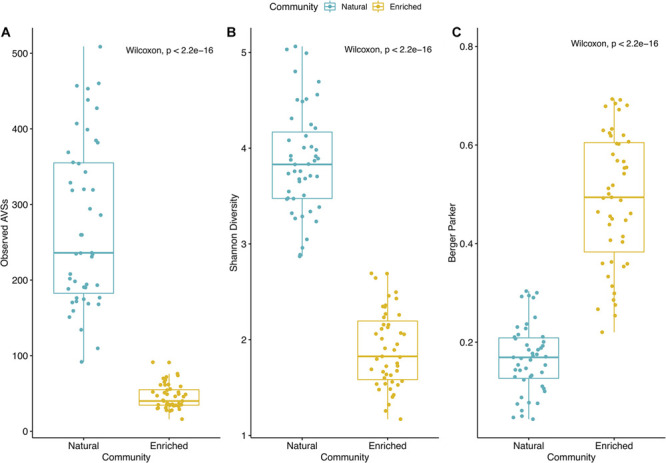
Alpha diversity indexes for natural and enriched microbial communities. **(A)** Number of observed ASVs; **(B)** Shannon diversity index; **(C)** Berger parker index. Each sample is represented by one point. The boxes represent the first and third quartiles, with median value bisecting each box. The whiskers extend to the largest/smallest value.

**FIGURE 4:**
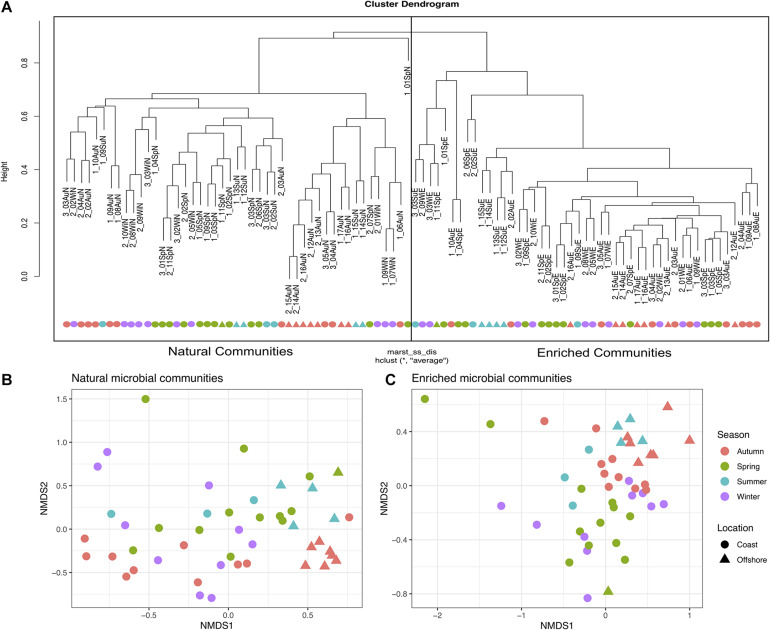
Beta diversity analysis performed for natural and enriched microbial communities. **(A)** Bray-Curtis hierarchical cluster of the natural and enriched communities of all 47 sampled sites [the first number represents the sampling area (1, 2, or 3)]. **(B)** Non-metric multidimensional scaling (NMDS) for all 47 natural microbial communities, based on absolute abundance of ASVs, in relation to Season and Location. **(C)** NMDS for all 47 enriched microbial communities, based on absolute abundance of ASVs in relation to Season and Location.

The biodiversity present in the natural microbial communities significantly (*p* < 2.2e^−16^) decreased in richness (Number of observed ASVs – Figure 3A) and diversity (Shannon index – Figure 3B), after the 2-week enrichment with crude-oil. Furthermore, the enrichment communities were significantly (*p* < 2.2e^−16^) dominated by the most abundant ASVs in comparison to natural microbial communities (Berger parker index – Figure 3C). In agreement, hierarchical cluster analysis shows a clear distinction between natural microbial communities and oil-enriched microbial communities (ANOSIM: *R* = 0.961; *p* < 0.001; Figure 4A). This analysis showed that the parameters sampling area (1, 2, and 3), location (coast, offshore), and season (Sp, Su, Au, and Wi) seem to have no effect in establishing differences in community patterns, when natural and enriched samples are analyzed together. However, when non-metric multidimensional scaling (NMDS) analysis was applied individually for natural (Figure 4B) and enriched (Figure 4C) samples, results showed that prokaryotic communities collected in different seasons are significantly different (ANOSIM: *R* = 0.134; *p* < 0.01 – for natural samples; ANOSIM: *R* = 0.243; *p* < 0.001 – for enriched samples). In addition, this analysis revealed that these prokaryotic communities are highly homogeneous in terms of sampling area (1, 2, and 3) and location (coast, offshore).

### Taxonomic Profiles in Natural and Crude Oil Enrichment Samples

#### Relevant Phyla

**FIGURE 5:**
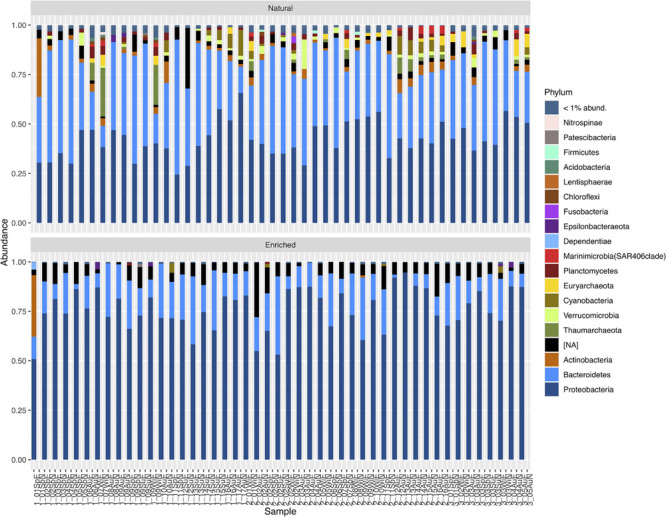
Taxonomic profile of relative abundances of ASVs of natural and enriched microbial communities of 47 sampled sites, at the highest taxonomic level (Phyla with relative abundances below 1% were not considered). NA, Not Assigned.

Natural prokaryotic community characterization at the highest taxonomic level showed that these communities presented high relative abundances for the phyla Bacteroidetes (between approximately 11 and 68%), Proteobacteria (≈ 24–66%), Actinobacteria (≈ 0.23–30%), Thaumarchaeota (≈ 0–25%), Verrucomicrobia (≈ 0.06–15%), Cyanobacteria (≈ 0.02–13%), and Euryarchaeota (≈ 0–9%) (Figure 5). In the enriched samples, the selected prokaryotic consortia were composed predominately by Proteobacteria (approximately between 51 and 95%) and Bacteroidetes (≈ 0.15–40%). Oil-enriched communities showed high relative abundance of not assigned sequences at the phylum level (Figure 5), in comparison to natural microbial communities. However, by observing the richness of not identified ASVs at phylum level, results show that in the natural microbial communities the number of not assigned ASVs varied between 19 and 244 (average of 92) and in oil-enriched communities it varied between 16 and 196 (average of 88).

#### Potential Hydrocarbon-Degrading Prokaryotes

Of the 1643 distinct taxa identified in the natural and oil-enriched microbial communities, combined, only 55 taxa were found to be equally abundant in both communities, 841 taxa were predominant in the natural communities and only 106 taxa were more abundant in the enriched communities.

**FIGURE 6:**
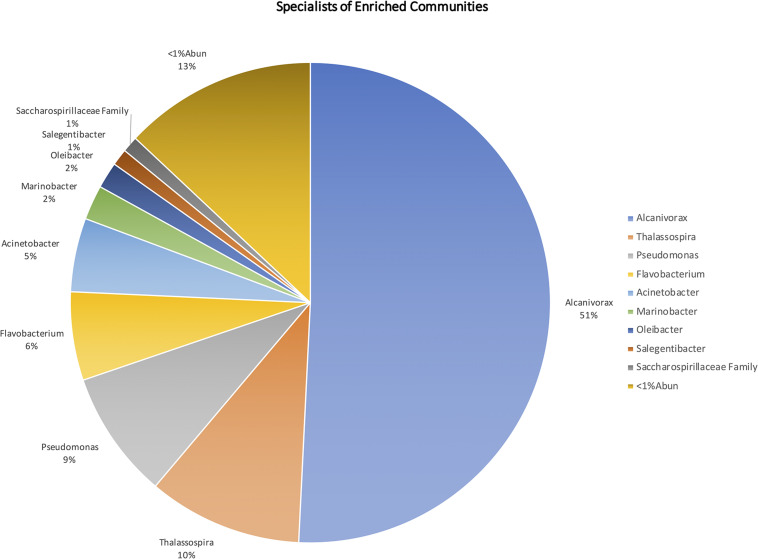
Microorganisms present in the enriched microbial communities, at genus level, with relative abundance >1% in relation to the total enriched microorganisms.

Specialist analysis allowed to distinguish a group of genera that were significantly more abundant in the oil-enriched communities (Figure 6), revealing that these communities were dominated by genera known as HC degraders, such as *Alcanivorax*, *Thalassospira*, *Pseudomonas*, *Marinobacter*, *Alkanindiges*, *Alteromonas*, *Cycloclasticus*, *Halomonas*, *Joostella*, *Marinomonas*, *Rhodococcus*, and *Thalassolituus*. Most of these genera were also detected in the natural communities but in very low number of ASVs. Moreover, a few genera known HC degraders were found mainly in the natural communities (e.g., *Colwellia*, *Oleiphilus*, *Oleispira*, and *Ulvibacter*), while some others were considered equally abundant in both natural and enriched communities (e.g., *Polaribacter*, *Pseudoalteromonas*, and *Roseobacter clade NAC11-7 lineage*).

#### Relevant Genera

**FIGURE 7:**
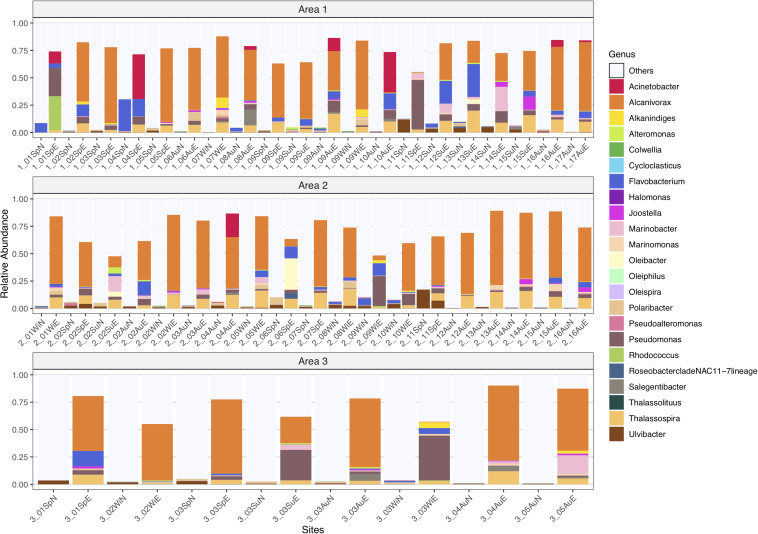
Taxonomic profile of relative abundances of ASVs for natural prokaryotic communities and oil-enriched communities, in 47 sampled sites, distributed per Area (1, 2, and 3), for 23 genera known to degrade petroleum hydrocarbons.

The analysis of the distribution of the lower taxonomic level groups in all samples was performed for the 23 genera known to degrade petroleum hydrocarbons. Overall, there was a great increase in abundance of HC degrading genera after the 15-day crude-oil enrichment, such as in the OHCB *Alcanivorax* (0.04 to ≈ 70%) and *Marinomonas* (0.64 to ≈ 5.9%), as well as in other genera like *Acinetobacter* (0.60 to ≈ 41%), *Joostella* (0.26 to ≈ 12%), *Oleibacter* (0.15 to ≈ 28%), *Pseudomonas* (0.76 to ≈ 45%), *Rhodococcus* (0.04% increased to ≈ 31%), *Salegentibacter* (2.81 to ≈ 14%), and *Thalassospira* (0.01 to ≈ 20%) (Figure 7).

Nonetheless, some HC-degrading genera that were present in the natural microbial communities decreased in abundance in the enriched prokaryotic communities, like the OHCB *Oleiphilus* (0.09 to 0%) and *Oleispira* (0.25 to 0.08%), and also *Ulvibacter* (17 to ≈ 7%). *Flavobacterium* was the only genus that kept its abundance of ≈30% in both communities.

### Predicted Functional Profile

**FIGURE 8:**
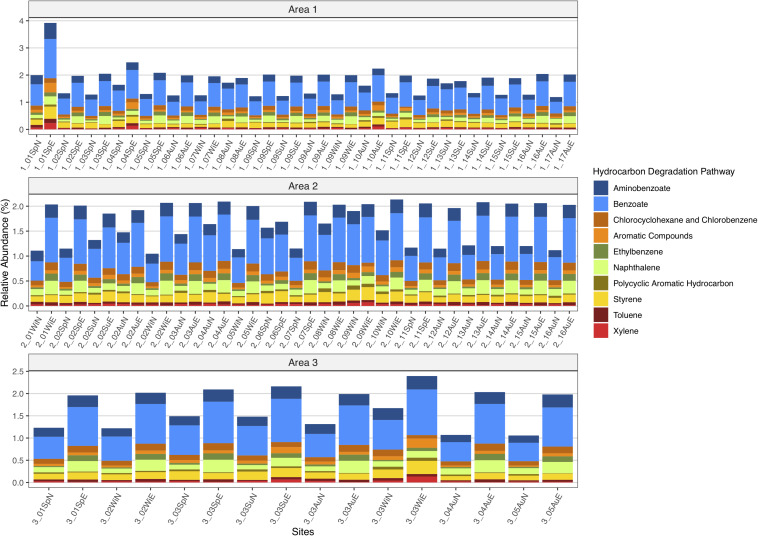
Predicted relative abundance of genes involved in ten petroleum hydrocarbon degradation metabolisms present in the natural microbial communities and in oil-enriched communities, for the 47 sampled sites, distributed per Area (1, 2, and 3).

The predicted functional profiling of the microbial communities showed that the natural communities were equipped with machinery to degrade petroleum compounds (Figure 8) and after the oil-enrichment there was an overall increase in abundance of predicted KOs known to be involved in hydrocarbons degradation (Figure 8).

Regarding the ten HC degradation pathways analyzed for the 47 sampled sites, there was an increase, on average, in the capability to degrade aminobenzoate (≈ 38%) benzoate (≈ 58%), chlorocyclohexane and chlorobenzene (≈ 37%), aromatic compounds (≈ 59%), ethylbenzene (≈ 197%), naphthalene (≈ 115%), styrene (≈ 10%), and toluene (≈ 64%) from natural microbial communities to enriched microbial communities. However, there was an average decrease in capability to degrade PAH (−27%) and xylene (−2%) compounds.

#### Taxonomic Contributions *per* Metabolism

**FIGURE 9:**
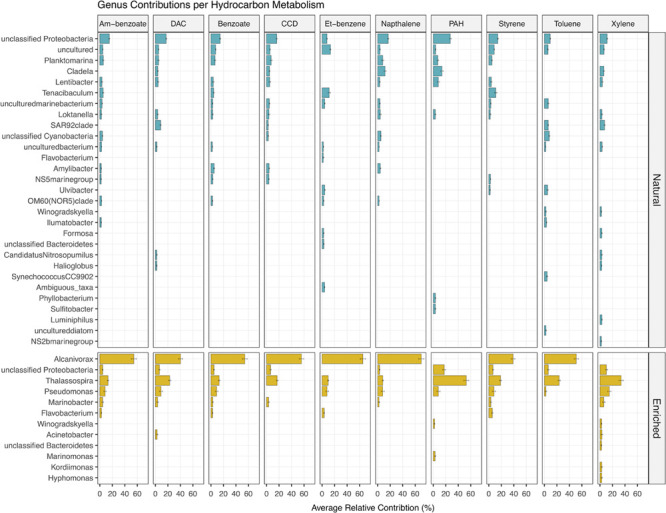
Prediction of the relative contribution (above 2%) of each genus for the 10 aromatic hydrocarbon degradation pathways present in natural and oil-enriched microbial communities. Am-benzoate, Aminobenzoate; DAC, Degradation of Aromatic Compounds; CCD, Chlorocyclohexane and Chlorobenzene Degradation; Et-benzene, Ethylbenzene.

In the natural communities, it was predicted that a high number of different taxa (841 genera) contributed to the potential capability to degrade different petroleum aromatic HCs (Figure 9). However, the relative contribution of each individual genus did not surpass ≈11%, which was the highest relative contribution observed for *Tenacibaculum* for ethylbenzene degradation. On the other hand, in the enriched communities, it was observed a lower number of different taxa (217 genera) contributing to the potential capability to degrade different petroleum aromatic HCs, where a distinct contribution of only five taxa was observed for all 10 degradation metabolisms, namely *Alcanivorax*, *Thalassospira*, *Pseudomonas*, *Marinobacter*, and *Flavobacterium* (Figure 9). The cumulative contribution of these genera corresponded to more than 50% of the relative contribution to the degradation of the 10 different petroleum HCs. In enriched communities *Alcanivorax* was the genus that had higher relative contributions in all metabolisms (varying between ≈39 and ≈70%), with the exception for PAH and xylene degradation with a 0% contribution in both pathways.

### Abundance of Hydrocarbon Degraders Estimated by MPN

The MPN analysis confirmed that, overall, there was an increase in abundance of hydrocarbon-degrading microorganisms during the enrichment period for all sampled sites (Supplementary Table 3). Results indicate that both native and enriched microbial communities had the ability to degrade petroleum HCs. Although some natural communities presented values below the detection limit, the abundance of hydrocarbon-degrading microorganisms sharply increased after the crude-oil enrichments.

## Discussion

In the present work, the distribution and selection of native hydrocarbon-degrading microorganisms at different sites along the NW coast of the Iberian Peninsula were investigated.

Enrichment experiments allowed the identification and characterization of native microbial consortia with the ability to degrade petroleum compounds, during the early stages of the bioremediation of oil spills.

Rarefaction curves showed that the biodiversity present in natural microbial communities decreased due to the oil-enrichment experiments, which is in agreement with the lower values of Shannon diversity index registered in the oil-enriched communities. Since petroleum is composed of recalcitrant and highly toxic compounds, it is expected that crude-oil will induce a selective pressure onto the natural microbial communities and only a small fraction of microorganisms capable of surviving and/or degrading petroleum HCs were able to adapt. This also explains why fewer dominant ASVs account for most of the total number of individuals in the enriched communities, as stated by the Berger-Parker index. Similar results were obtained in a study conducted after the Deepwater Horizon oil spill, where a significant decrease in diversity was observed in oil-plume communities when comparing with non-oil plume communities in the deep-sea (Redmond and Valentine, 2012). Ribicic et al. (2018) also observed a decrease in species richness and Shannon diversity indexes in microcosms experiments with oil and dispersant incubated for 64 days with natural seawater collected at a Norwegian fjord.

Prokaryotic communities from oil contaminated marine sediments have been previously described has being dominated by Proteobacteria, Bacteroidetes, and Actinobacteria. The dominance of these phyla has been registered in beaches from the northern Spanish coast affected by the Prestige (Acosta-González et al., 2015) as well as in a coastal salt marsh affected by the Deepwater Horizon spill (Beazley et al., 2012). In addition, this last study revealed that once HCs became undetected, the abundance of these phyla decreased, showing a clear relationship between the presence of oil in the sediments and the dominance of the Proteobacteria, Bacteroidetes, and Actinobacteria. In agreement, our experiments showed that after the 2-week enrichments, the native microbial communities were mainly composed of the phylum Proteobacteria, with a relative abundance ranging between 51 and 95%, followed by Bacteroidetes with a relative abundance between 0.15 and 40%. The results obtained in this study, regarding phyla, indicate that a clear selection of native microbial consortia capable of degrading petroleum hydrocarbons occurred.

A considerable high number of ASVs were not identified at phylum level in the oil-enriched samples, suggesting that our experimental approach was useful to select new HC degradation competent prokaryotes that are naturally available but difficult to cultivate in laboratory. Future research on these unidentified sequences may reveal novel taxa that may represent a high fraction of the still unknown natural microbial diversity able to degrade HC (Pascoal et al., 2020).

When an oil spill happens, there is a high input of hydrocarbons in the environment and there are not enough nutrients, such as nitrogen and phosphorous, to support the microbial growth requirements. Adding these nutrients in enrichment experiments contaminated with petroleum should stimulate the growth of HC-degrading microorganisms (Harayama et al., 2004), which was the approach used in this study. The oil-enriched microbial communities were mainly dominated by one of the known OHCB genus, *Alcanivorax* (up to 70%). This observation is consistent with previous studies where seawater containing crude-oil or heavy oil, supplemented with nitrogen and phosphorous fertilizers, were dominated by *Alcanivorax* (Kasai et al., 2002b). Also, for beach sediments contaminated with oil, treated with inorganic nutrients and using synthetic water, the bacterial community was dominated by *Alcanivorax* (Röling et al., 2002).

*Pseudomonas* (up to 45%) and *Flavobacterium* (up to 30%) were also found to be highly represented genera in the oil-enriched communities in this study. Kaplan and Kitts (2004) observed that these two genera were associated with the rapid degradation of total petroleum hydrocarbons of a land treatment unit contaminated with weathered petroleum HCs, supplemented with nutrients, during the first 3 weeks. *Flavobacterium* also had a relative high abundance in the natural communities (up to 29%), however, some strains in this genus are also described as being capable to mineralize several types of organic matter in aquatic ecosystems (Bernardet and Bowman, 2006). Due to the selective pressure that these communities were under by the 2-week incubation period with crude-oil, it is possible to assume that the presence of *Flavobacterium* in the enriched communities may indicate that this genus contains members capable of degrading petroleum HCs. This is corroborated by previous studies showing the degradation potential of some petroleum HCs by *Flavobacterium* spp. (Rahman et al., 2003; Chaudhary and Kim, 2018). Regarding *Acinetobacter*, this genus had a variation from 0 to 41% in relative abundance in the enriched communities. A study conducted using hydrocarbon-degrading microorganisms isolated from soil samples contaminated with petroleum, from the Lingala Oil field Project, in India, showed that a strain of *Acinetobacter calcoaceticus* had a similar performance in degradation of alkanes and aromatic HCs as a strain of *Pseudomonas* that could degrade around 70% of alkanes and 45% of aromatics (Mittal and Singh, 2009). *Rhodococcus* had a relative abundance of up to 31% in the crude-oil enriched communities. In fact, Li et al. (2013) showed that the strain *Rhodococcus* sp. JZX-01 could degrade up to 65% of long-chain HCs and branched alkanes present in crude-oil in 9 days. In addition, the species *Rhodococcus soli*, isolated from sediment samples from an oil-contaminated beach in Korea, was found to have a significant impact in the removal of PAHs (Lee et al., 2018).

*Oleibacter* was found in the enriched communities with a relative abundance varying between 0 and 28%. This genus contains the hydrocarbonoclastic strain *Oleibacter marinus* that degrades aliphatic HCs (Teramoto et al., 2011). Lofthus et al. (2018) evaluated the biodegradation of n-alkanes by microbial communities present in seawater from a Norwegian fjord, with a hydrophobic adsorbent system at different temperatures and found that *Oleibacter* was the most abundant genus at 20°C. The OHCB genus *Marinobacter* was also found in the enriched communities, with relative abundances between 0 and 22%. This genus was also found in a Florida’s beach sand exposed to heavy petroleum contamination from the Deep Water Horizon, through both cultivation-dependent and independent methodologies, portraying a key role in the degradation of petroleum compounds together with *Alcanivorax* (Kostka et al., 2011). *Thalassospira* is also a genus that has some species described with the potential to degrade petroleum hydrocarbons such as *Thalassospira tepidiphila* that can degrade polycyclic aromatic compounds, like naphthalene, phenanthrene, and fluorene (Kodama et al., 2008). In the present study, this genus accounted for a relative abundance of 0.5–20% in the oil-enriched communities. Also present in the crude-oil enriched communities, with a relative high abundance, were the genera *Salegentibacter* (up to 14%), *Joostella* (up to 12%) and *Alkanindiges* (up to 10%), previously identified as hydrocarbon-degraders (Bogan et al., 2003; Hassanshahian and Boroujeni, 2016; Rizzo et al., 2018).

The fact that genera present in the oil-enriched communities were not detected or had low abundance in the natural microbial communities, and their abundance increased after the 2-week petroleum enrichment, indicated that these prokaryotes composed the natural microbial rare biosphere, acting as “seed banks” until the appropriate conditions, with the right nutrient proportions, were present for them to increase their abundance (Cerqueda-García et al., 2020; Pascoal et al., 2020).

On the other hand, some OHCB genera that were present in natural microbial communities, such as *Cycloclasticus* (up to 0.02%), *Oleispira* (up to 0.25%), and *Oleiphilus* (up to 0.09%), were not enhanced by the oil-enrichment experiments performed in the present study. These points to some constrain in the enrichment process developed in the present study. For example, some restraints in enhancing the abundance of *Cycloclasticus* might have occurred since bacteria from this genus are specialized in degrading more complex HC (Kasai et al., 2002a). Since the enrichment experiments were only performed for 2 weeks, maybe the biodegradation rate of petroleum compounds did not reach the HC more difficult to degrade and the lack of specific substrates inhibited its growth. Also, the petroleum used in this study was Arabian light crude oil which contains more aliphatic compounds and low proportion of aromatics (Cerqueda-García et al., 2020). Regarding the lack of increase in *Oleispira* after crude-oil enrichment experiments, it might be due to the temperature at which the enrichment flasks were exposed (20°C), since an increase of microorganisms from this genus was only observed for microcosms maintained at 4°C (Coulon et al., 2007).

The predicted functional profile of natural and crude-oil enriched microbial communities showed that both had the potential to degrade aromatic hydrocarbons, such as aminobenzoate, benzoate, chlorocyclohexane, chlorobenzene, ethylbenzene, naphthalene, PAHs, styrene, toluene, and xylene, based on KOs abundance known to encode enzymatic mechanisms involved in the degradation of these compounds. Moreover, there was an increase in abundance of KOs in most of the degradation metabolisms after 2 weeks of enrichment with crude oil, with the exception of PAHs and xylene degradation metabolisms. These results are in agreement with the increase in abundance of microorganisms that possess the metabolic pathways to degrade different aromatic HCs obtained in this work, as described above. The correlation between the abundance of certain KOs with the presence of different microorganisms known to degrade different types of HCs, was observed in a study comprising NGS data obtained from different experiments performed on different environments and matrixes polluted with oil HCs (Mukherjee et al., 2017). In addition, Chikere et al. (2019) observed that regardless the geographical location and environmental conditions, when petroleum is introduced in soil matrixes it sets off a genetic selection for native HC-degrading microorganisms with the metabolic capacity to biodegrade petroleum compounds. Other studies have also analyzed the correlation between the efficiency of biodegradation of petroleum HCs with the relative abundance of genes associated with contaminant removal (Tao et al., 2017; Roy et al., 2018; Neethu et al., 2019). Although PICRUSt2 gives only a prediction of the functional profile, the results obtained from the taxa contributions for each degradation pathway showed that genera that had an increase in abundance during enrichments, such as *Alcanivorax*, *Thalassospira*, *Pseudomonas*, and *Marinomonas*, contributed to the increase in the potential to degrade aromatic compounds. Moreover, one can observe that the decrease in relative abundance of KOs involved in the capability to degrade PAHs and xylene might be due to *Alcanivorax* not being equipped with machinery to do so, since the contribution of this genus was nule in both degradation pathways.

Toward the development of an efficient hydrocarbon-degrading consortium, a shotgun metagenomics analysis should be further applied to fully understand which genes are present in the native microbial communities and select microorganisms with higher bioremediation efficiency.

In this work, the hydrocarbon-degrading microbial abundance was estimated by the MPN method, for both natural and oil-enriched microbial communities, showing that all sampled sites present capacity to degrade petroleum hydrocarbons. Despite the low abundances obtained (ranging between 0 and 2.67 ×10^11^ MPN/mL), natural microbial communities had hydrocarbon degrading microorganisms in their composition, so it was expected that by the end of the crude-oil enrichments their abundance would increase, as it was observed. These results are corroborated by Medina-Bellver et al. (2005), who also obtained an increase in the values of MPN, specifically for degraders of the aromatics undecane, naphthalene and phenanthrene, in seawater samples collected 1 month after the Prestige oil spill. The results obtained in this work by the MPN method further confirmed that the developed workflow successfully selected native microbial consortia with the capability to degrade hydrocarbons.

When oil spills disasters occur, different native microorganisms are selected according to the bioavailability of different hydrocarbons. After the oil spill, the native communities have the capacity to return to a composition similar to that initially present before the pollution event (Dubinsky et al., 2013). Therefore, the use of native microbial consortia to bioremediate an oil spill will help to accelerate the process of selection and succession, that already naturally happens as a response to the stress induced by the contaminant. Afterward the microbial communities may have the capacity to return to its pre-oil composition due to the high resilience capacity of microbial communities. Overall, the combined action of different microorganisms capable of degrading petroleum compounds is the best approach to mitigate oil spills, since different organisms can biodegrade different classes of hydrocarbons (Varjani, 2017). The aim of using native microbial consortia capable of degrading petroleum compounds is to avoid the introduction of exogenous microorganisms and to increase the bioremediation efficiency by using organisms already adapted to the environmental conditions of the oil-contaminated site. Thus, the application of native microbial consortia as a bioremediation approach will provide an eco-friendly, efficient and economic technique to mitigate oil spill disasters, that will also allow restoring ecosystems functions.

## Conclusion

Microorganisms with the capability to degrade petroleum hydrocarbons were found widely distributed along the NW coast of the Iberian Peninsula and it was possible to successfully select them to identify native microbial consortia with the potential to degrade the first stages of petroleum hydrocarbons. The enriched HC-degrading communities were mainly composed by *Alcanivorax* spp., *Pseudomonas* spp., *Acinetobacter* spp., *Rhodococcus* spp., *Flavobacterium* spp., *Oleibacter* spp., *Marinobacter* spp., and *Thalassospira* spp. The predicted functional profiling indicated that these enriched communities have the potential to degrade some aromatic compounds, such as aminobenzoate, benzoate, chlorocyclohexane, chlorobenzene, ethylbenzene, naphthalene, PAHs, styrene, toluene, and xylene.

This study identified several native key-microorganisms capable to biodegrade petroleum compounds, highly relevant to implement an efficient culturing work flow to recover bioremediation consortia. Moreover, this study also proved that, at a genera level, the 47 enriched communities have no significant differences among them which could indicate that we can have a prototype consortium of hydrocarbon-degrading bacteria to be used along the NW coast of the Iberian Peninsula.

Further studies should involve the recovering of the selected non-identified prokaryotes that respond to petroleum addition that represents a significant fraction of the enrichment communities, using culture-dependent combined with metagenomic approaches. In addition, analysis at metagenomic level would be beneficial to study the potential functional capabilities of the oil-enriched communities to degrade hydrocarbons.

## Data Availability Statement

The datasets presented in this study can be found in online repositories. The names of the repository/repositories and accession number(s) can be found below: European Nucleotide Archive, accession no: PRJEB43289 (ACCESSION PRIVATE) https://www.ebi.ac.uk/ena/browser/view/PRJEB43289.

## Author Contributions

CM, AB, CA, and AM: conceptualization. MB, RP, DA, JF, AB, and CA: laboratory and field work. MB, MS, CM, and AM: data curation. MB, CM, and AM: writing – original draft preparation. MB, CM, RP, DA, JF, AB, SR, MC, MS, JL, CA, and AM: writing – review and editing. CM, JL, and AM: supervision. CM, SR, MC, CA, and AM: project administration and funding acquisition. All authors have read and agreed to the published version of the manuscript.

## Conflict of Interest

The authors declare that the research was conducted in the absence of any commercial or financial relationships that could be construed as a potential conflict of interest.

## Footnotes

1 https://www.ebi.ac.uk/ena/browser/view/PRJEB43289

## Abbreviations

HC: Hydrocarbon
PAH: Polycyclic Aromatic Compounds
NGS: Next-Generation Sequencing
OHCB: Obligate Hydrocarbonoclastic Bacteria
rRNA: ribosomal RNA
PCR: Polymerase Chain Reaction
ASV: Amplicon Sequence Variants
MPN: Most Probable Number.
