# Supplementary material for: Harnessing the Potential of Native Microbial Communities for Bioremediation of Oil Spills in the Iberian Peninsula NW Coast
Source: Front Microbiol. 2021 Apr 23;12:633659. doi: 10.3389/fmicb.2021.633659 (PMC8102992; doi:10.3389/fmicb.2021.633659)
Supplement: Supplementary file 1 [file Table_1.docx]

Supplementary Material

Supplementary Table 1. Characterization of the sites sampled along the NW coast of the Iberian Peninsula. Su – Summer, Au – Autumn, Wi – Winter, Sp – Spring, N – Natural, E – Enriched.

| **Area** | **Station** | **Sampling Site** | **Season** | **Collection Date** | **Latitude** | **Longitude** | **Community Type** | | |
| --- | --- | --- | --- | --- | --- | --- | --- | --- | --- |
|  |  |  |  |  |  |  | **Natural** | **Enriched** | |
| **1** | 1_01 | Caminha | Spring | 30/05/2018 | 41º52'47.72" N | 8º51'49.00" W | 1_01SpN | | 1_01SpE |
| **1** | 1_02 | Vila Praia de Âncora | Spring | 30/05/2018 | 41º49'34.24" N | 8º52'24.59" W | 1_02SpN | | 1_02SpE |
| **1** | 1_03 | Carreço | Spring | 30/05/2018 | 41º44'31.26" N | 8º52'38.61" W | 1_03SpN | | 1_03SpE |
| **1** | 1_04 | Lima Estuary | Spring | 13/06/2018 | 41º40'36.69" N | 8º49'45.90" W | 1_04SpN | | 1_04SpE |
| **1** | 1_05 | São Bartolomeu do Mar | Spring | 13/06/2018 | 41º33'56.6" N | 8º47'47.8" W | 1_05SpN | | 1_05SpE |
| **1** | 1_06 | Vila Chã | Winter | 30/01/2018 | 41º17'44.736" N | 8º44'13.74" W | 1_06AuN | | 1_06AuE |
| **1** | 1_07 | Cabo do Mundo | Winter | 30/01/2018 | 41º13'10.56" N | 8º42'54.23" W | 1_07WiN | | 1_07WiE |
| **1** | 1_08 | Leixões | Autumn | 20/09/2017 | 41º10'38.20" N | 8º42'7.45" W | 1_08AuN | | 1_08AuE |
| **1** | 1_09 | Ingleses | Autumn | 20/09/2017 | 41º9'1.42" N | 8º40'37.05" W | 1_09AuN | | 1_09AuE |
| **1** | 1_09 | Ingleses | Spring | 07/05/2018 | 41º9'1.42" N | 8º40'37.05" W | 1_09SpN | | 1_09SpE |
| **1** | 1_09 | Ingleses | Summer | 12/07/2018 | 41º9'1.42" N | 8º40'37.05" W | 1_09SuN | | 1_09SuE |
| **1** | 1_09 | Ingleses | Winter | 30/01/2018 | 41º9'1.42" N | 8º40'37.05" W | 1_09WiN | | 1_09WiE |
| **1** | 1_10 | Cabedelo | Autumn | 20/09/2017 | 41º8'30.00" N | 8º39'58.38" W | 1_10AuN | | 1_10AuE |
| **1** | 1_11 | Offshore | Spring | 07/05/2018 | 41º32'22.632" N | 8º51'54.36" W | 1_11SpN | | 1_11SpE |
| **1** | 1_12 | Offshore | Summer | 26/07/2018 | 41º36'6.048" N | 8º52'8.292" W | 1_12SuN | | 1_12SuE |
| **1** | 1_13 | Offshore | Summer | 26/07/2018 | 41º29'0.78" N | 8º49'31.44" W | 1_13SuN | | 1_13SuE |
| **1** | 1_14 | Offshore | Summer | 20/08/2018 | 41º36.465N | 8º48.975W | 1_14SuN | | 1_14SuE |
| **1** | 1_15 | Offshore | Summer | 20/08/2018 | 41º37.689N | 8º51.696W | 1_15SuN | | 1_15SuE |
| **1** | 1_16 | Offshore | Autumn | 28/09/2018 | 41°14'30.0" N | 9°16'30.0" W | 1_16AuN | | 1_16AuE |
| **1** | 1_17 | Offshore | Autumn | 28/09/2018 | 41°07'18.0" N | 9°18'28.2" W | 1_17AuN | | 1_17AuE |
| **2** | 2_01 | Oia | Winter | 15/02/2018 | 42º0'3.24" N | 8º52'37.931" W | 2_01WiN | | 2_01WiE |
| **2** | 2_02 | Toralla | Autumn | 18/10/2017 | 42º12'3.6" N | 8º47'57.48" W | 2_02AuN | | 2_02AuE |
| **2** | 2_02 | Toralla | Spring | 19/04/2018 | 42º12'3.6" N | 8º47'57.48" W | 2_02SpN | | 2_02SpE |
| **2** | 2_02 | Toralla | Summer | 06/07/2017 | 42º12'3.6" N | 8º47'57.48" W | 2_02SuN | | 2_02SuE |
| **2** | 2_02 | Toralla | Winter | 15/02/2018 | 42º12'3.6" N | 8º47'57.48" W | 2_02WiN | | 2_02WiE |
| **2** | 2_03 | Cesantes | Autumn | 18/10/2017 | 42º18'39.24" N | 8º37'14.16" W | 2_03AuN | | 2_03AuE |
| **2** | 2_04 | Rodeira | Autumn | 18/10/2017 | 42º15'40.68" N | 8º46'9.48" W | 2_04AuN | | 2_04AuE |
| **2** | 2_05 | A Lanzada | Winter | 15/02/2018 | 42º26'59.64" N | 8º52'40.8" W | 2_05WiN | | 2_05WiE |
| **2** | 2_06 | A Corna | Spring | 15/05/2018 | 42º35'6.72" N | 8º57'3.6" W | 2_06SpN | | 2_06SpE |
| **2** | 2_07 | Corrubedo | Spring | 15/05/2018 | 42º34'27.48" N | 9º3'2.52" W | 2_07SpN | | 2_07SpE |
| **2** | 2_08 | Do Cruceiro | Winter | 07/03/2018 | 42º43'35.76" N | 9º0'2.16" W | 2_08WiN | | 2_08WiE |
| **2** | 2_09 | Broña | Winter | 07/03/2018 | 42º48'5.76" N | 8º55'44.04" W | 2_09WiN | | 2_09WiE |
| **2** | 2_10 | Reimunde | Winter | 07/03/2018 | 42º46'53.76" N | 8º59'48.12" W | 2_10WiN | | 2_10WiE |
| **2** | 2_11 | Carnota | Spring | 19/04/2018 | 42º50'8.52" N | 9º6'21.6" W | 2_11SpN | | 2_11SpE |
| **2** | 2_12 | Offshore | Autumn | 23/11/2017 | 42º5'2.605" N | 8º57'0.58" W | 2_12AuN | | 2_12AuE |
| **2** | 2_13 | Offshore | Autumn | 23/11/2017 | 42º8'32.435" N | 9º2'16.289" W | 2_13AuN | | 2_13AuE |
| **2** | 2_14 | Offshore | Autumn | 27/11/2017 | 42º8'22.2" N | 8º57'34.798" W | 2_14AuN | | 2_14AuE |
| **2** | 2_15 | Offshore | Autumn | 27/11/2017 | 42º12'32.4" N | 8º58'8.4" W | 2_15AuN | | 2_15AuE |
| **2** | 2_16 | Offshore | Autumn | 27/11/2017 | 42º8'28.201" N | 8º54'24.602" W | 2_16AuN | | 2_16AuE |
| **3** | 3_01 | Nemiña | Spring | 19/04/2018 | 43º0'23.76" N | 9º15'35.28" W | 3_01SpN | | 3_01SpE |
| **3** | 3_02 | Razo | Winter | 13/03/2018 | 43º17'29.04" N | 8º42'6.84" W | 3_02WiN | | 3_02WiE |
| **3** | 3_03 | Caranza | Autumn | 09/11/2017 | 43º28'38.28" N | 8º12'9" W | 3_03AuN | | 3_03AuE |
| **3** | 3_03 | Caranza | Spring | 15/05/2018 | 43º28'38.28" N | 8º12'9" W | 3_03SpN | | 3_03SpE |
| **3** | 3_03 | Caranza | Summer | 07/07/2017 | 43º28'38.28" N | 8º12'9" W | 3_03SuN | | 3_03SuE |
| **3** | 3_03 | Caranza | Winter | 13/03/2018 | 43º28'38.28" N | 8º12'9" W | 3_03WiN | | 3_03WiE |
| **3** | 3_04 | A Frouxeira | Autumn | 09/11/2017 | 43º36'38.16" N | 8º10'17.04" W | 3_04AuN | | 3_04AuE |
| **3** | 3_05 | Morouzos | Autumn | 09/11/2017 | 43º42'10.976" N | 7º50'35.336" W | 3_05AuN | | 3_05AuE |
