# Supplementary material for: Harnessing the Potential of Native Microbial Communities for Bioremediation of Oil Spills in the Iberian Peninsula NW Coast
Source: Front Microbiol. 2021 Apr 23;12:633659. doi: 10.3389/fmicb.2021.633659 (PMC8102992; doi:10.3389/fmicb.2021.633659)
Supplement: Supplementary file 2 [file Table_2.docx]

Supplementary Material

Supplementary Table 2. Number of available sequences present in Ilumina libraries (Raw sequences) and after DADA2 pipeline (Filtered Sequences), in Natural (N) and Enriched (E) communities.

| **Station** | **Raw Reads** | **Filtered Sequences** |  | **Station** | **Raw Reads** | **Filtered Sequences** |
| --- | --- | --- | --- | --- | --- | --- |
| **1_01SpN** | 49630 | 26919 |  | **1_01SpE** | 38799 | 22665 |
| **1_02SpN** | 68670 | 40899 |  | **1_02SpE** | 32054 | 16762 |
| **1_03SpN** | 52990 | 32874 |  | **1_03SpE** | 83013 | 41634 |
| **1_04SpN** | 64469 | 40944 |  | **1_04SpE** | 68012 | 42979 |
| **1_05SpN** | 59240 | 37267 |  | **1_05SpE** | 35810 | 18021 |
| **1_06AuN** | 51002 | 20793 |  | **1_06AuE** | 47857 | 30136 |
| **1_07WiN** | 75880 | 43238 |  | **1_07WiE** | 58385 | 37794 |
| **1_08AuN** | 95091 | 69124 |  | **1_08AuE** | 52493 | 37713 |
| **1_09SpN** | 65252 | 41273 |  | **1_09SpE** | 33838 | 19582 |
| **1_09SuN** | 43914 | 21556 |  | **1_09SuE** | 31822 | 17313 |
| **1_09AuN** | 57902 | 31919 |  | **1_09AuE** | 82185 | 55858 |
| **1_09WiN** | 82642 | 50257 |  | **1_09WiE** | 64404 | 39411 |
| **1_10AuN** | 73120 | 45246 |  | **1_10AuE** | 88677 | 65092 |
| **1_11SpN** | 75159 | 48006 |  | **1_11SpE** | 58598 | 33259 |
| **1_12SuN** | 87747 | 58087 |  | **1_12SuE** | 61198 | 32413 |
| **1_13SuN** | 75613 | 47694 |  | **1_13SuE** | 39141 | 20505 |
| **1_14SuN** | 68254 | 47228 |  | **1_14SuE** | 34885 | 17513 |
| **1_15SuN** | 44532 | 30695 |  | **1_15SuE** | 34903 | 23461 |
| **1_16AuN** | 76202 | 48035 |  | **1_16AuE** | 95973 | 55110 |
| **1_17AuN** | 98388 | 62337 |  | **1_17AuE** | 54300 | 31574 |
| **2_01WiN** | 101197 | 62433 |  | **2_01WiE** | 31639 | 19123 |
| **2_02SpN** | 91766 | 55249 |  | **2_02SpE** | 54779 | 29503 |
| **2_02SuN** | 66965 | 37358 |  | **2_02SuE** | 61230 | 39222 |
| **2_02AuN** | 55957 | 33779 |  | **2_02AuE** | 65770 | 43535 |
| **2_02WiN** | 79645 | 45471 |  | **2_02WiE** | 36496 | 22488 |
| **2_03AuN** | 73934 | 49282 |  | **2_03AuE** | 55677 | 39589 |
| **2_04AuN** | 57309 | 36638 |  | **2_04AuE** | 57994 | 42354 |
| **2_05WiN** | 79203 | 50667 |  | **2_05WiE** | 53191 | 29907 |
| **2_06SpN** | 42759 | 24926 |  | **2_06SpE** | 108914 | 70978 |
| **2_07SpN** | 108760 | 72168 |  | **2_07SpE** | 86557 | 41836 |
| **2_08WiN** | 81583 | 48966 |  | **2_08WiE** | 49456 | 29951 |
| **2_09WiN** | 67925 | 43339 |  | **2_09WiE** | 73264 | 44942 |
| **2_10WiN** | 51706 | 32615 |  | **2_10WiE** | 58356 | 35153 |
| **2_11SpN** | 104731 | 67840 |  | **2_11SpE** | 60629 | 32323 |
| **2_12AuN** | 82378 | 54338 |  | **2_12AuE** | 59037 | 31866 |
| **2_13AuN** | 82467 | 53303 |  | **2_13AuE** | 42632 | 24027 |
| **2_14AuN** | 104108 | 72772 |  | **2_14AuE** | 49357 | 31430 |
| **2_15AuN** | 110024 | 76230 |  | **2_15AuE** | 59488 | 36533 |
| **2_16AuN** | 72316 | 51400 |  | **2_16AuE** | 69342 | 42704 |
| **3_01SpN** | 82004 | 46942 |  | **3_01SpE** | 40953 | 21556 |
| **3_02WiN** | 83445 | 56860 |  | **3_02WiE** | 65604 | 41020 |
| **3_03SpN** | 111066 | 70534 |  | **3_03SpE** | 45772 | 28301 |
| **3_03SuN** | 83278 | 57222 |  | **3_03SuE** | 44650 | 26956 |
| **3_03AuN** | 53943 | 27093 |  | **3_03AuE** | 68910 | 41782 |
| **3_03WiN** | 109014 | 59477 |  | **3_03WiE** | 83007 | 46791 |
| **3_04AuN** | 56020 | 34601 |  | **3_04AuE** | 58173 | 37833 |
| **3_05AuN** | 114733 | 76669 |  | **3_05AuE** | 55622 | 34528 |
