# Supplementary material for: Harnessing the Potential of Native Microbial Communities for Bioremediation of Oil Spills in the Iberian Peninsula NW Coast
Source: Front Microbiol. 2021 Apr 23;12:633659. doi: 10.3389/fmicb.2021.633659 (PMC8102992; doi:10.3389/fmicb.2021.633659)
Supplement: Supplementary file 3 [file Table_3.docx]

Supplementary Material

Supplementary Table 3. Abundance of hydrocarbon-degrading bacteria present in natural communities and enriched communities (mean values, standard deviations, n=3) evaluated by the Most Probable Number (MPN) method. a: no replicates, b: maximum detection limit value (> 8.0*10^11^ MPN/mL), c: values below the detection limit (<10MPN/mL) and d: unavailable information.

| **Sites** | **Natural Microbial Communities**  **(Log MPN/mL)** | **Enriched Microbial Communities**  **(Log MPN/mL)** |
| --- | --- | --- |
| **1_01Sp** | 1,82 ± 0,23 | 11,90*^a^* |
| **1_02Sp** | 5,27 ± 5,75 | 8,65 ± 1,34 |
| **1_03Sp** | 1,30 ± 0,42 | 11,903*^b^* |
| **1_04Sp** | 1,56 ± 0,19 | 11,61 ± 0,41 |
| **1_05Sp** | 1,18 ± 0,26 | 8,94 ± 2,83 |
| **1_06Au** | *c* | 11,903*^b^* |
| **1_07Wi** | 1,00 ± *a* | 11,903*^b^* |
| **1_08Au** | 2,60 ± 0,21 | 9,32 ± 4,48 |
| **1_09Sp** | *c* | 11,903*^b^* |
| **1_09Su** | 2,53 ± 0,19 | 11,90*^a^* |
| **1_09Au** | 2,54 ± 0,31 | 11,90 ± 0,01 |
| **1_09Wi** | 1,00*^a^* | 10,85 ± 1,82 |
| **1_10Au** | 2,82 ± 0,55 | 10,33 ± 2,18 |
| **1_11Sp** | *c* | 8,65 ± 0,14 |
| **1_12Su** | 1,44 ± 0,13 | 8,95 ± 2,90 |
| **1_13Su** | 2,25 ± 0,18 | 10,62 ± 1,28 |
| **1_14Su** | 2,17 ± 0,30 | 11,32 ± 1,01 |
| **1_15Su** | 2,98 ± 0,51 | 11,903*^b^* |
| **1_16Au** | 2,32 ± 0,22 | 8,37 ± 4,99 |
| **1_17Au** | 2,07 ± 0,15 | 11,903*^b^* |
| **2_01Wi** | 2,20*^a^* | 10,87 ± 1,78 |
| **2_02Sp** | 1,93 ± 0,70 | 9,48 ± 2,19 |
| **2_02Su** | *1,72±0,51* | 9,69 ± 2,79 |
| **2_02Au** | 2,16 ± 0,07 | 11,903*^b^* |
| **2_02Wi** | 2,54*^a^* | 11,24 ± 0,71 |
| **2_03Au** | 2,08*^a^* | 11,903*^b^* |
| **2_04Au** | 2,16 ± 0,07 | 11,903*^b^* |
| **2_05Wi** | 1,53*^a^* | 9,38 ± 1,41 |
| **2_06Sp** | *c* | 6,06 ± 1,02 |
| **2_07Sp** | 1,00*^a^* | 11,903*^b^* |
| **2_08Wi** | 2,99 ± 0,29 | 9,59 ± 2,05 |
| **2_09Wi** | 3,39 ± 0,08 | 11,71 ± 0,34 |
| **2_10Wi** | 2,22 ± 0,25 | 9,73 ± 2,64 |
| **2_11Sp** | *c* | 10,56 ± 1,85 |
| **2_12Au** | 1,24 ± 0,21 | 10,19 ± 1,92 |
| **2_13Au** | 1,00*^a^* | 11,903*^b^* |
| **2_14Au** | 1,96*^a^* | 11,903*^b^* |
| **2_15Au** | 1,00*^a^* | 11,903*^b^* |
| **2_16Au** | 1,32 ± 0,30 | 11,903*^b^* |
| **3_01Sp** | 1,42 ± 0,11 | 11,903*^b^* |
| **3_02Wi** | *d* | 8,98 ± 2,57 |
| **3_03Sp** | 1,37 ± 0,33 | 10,45 ± 2,52 |
| **3_03Su** | 1,59 ± 0,23 | 11,19 ± 1,24 |
| **3_03Au** | 2,08*^a^* | 11,903*^b^* |
| **3_03Wi** | *d* | 11,47 ± 0,75 |
| **3_04Au** | 1,42 ± 0,36 | 11,39 ± 0,89 |
| **3_05Au** | 1,18 ± 0,26 | 11,13 ± 1,09 |
